# Systems biology and artificial intelligence analysis highlights the pleiotropic effect of IVIg therapy in autoimmune diseases with a predominant role on B cells and complement system

**DOI:** 10.3389/fimmu.2022.901872

**Published:** 2022-09-30

**Authors:** Cristina Segú-Vergés, Silvia Caño, Elisabeth Calderón-Gómez, Helena Bartra, Teresa Sardon, Srini Kaveri, José Terencio

**Affiliations:** ^1^ Health Department, Anaxomics Biotech, Barcelona, Spain; ^2^ Grifols Innovation and New Technologies (GIANT) Ltd., Dublin, Ireland; ^3^ Medical Affairs, Grifols S.A., Sant Cugat del Vallés, Spain; ^4^ Institut National de la Santé et de la Recherche Médicale, Centre de Recherche des Cordeliers, Sorbonne Université, Université Paris Descartes, Sorbonne Paris Cité, Paris, France

**Keywords:** intravenous immunoglobulin, autoimmune diseases, inflammatory diseases, mathematical models, B cells, complement system, immunomodulation, IVIg Immunoglobulins

## Abstract

Intravenous immunoglobulin (IVIg) is used as treatment for several autoimmune and inflammatory conditions, but its specific mechanisms are not fully understood. Herein, we aimed to evaluate, using systems biology and artificial intelligence techniques, the differences in the pathophysiological pathways of autoimmune and inflammatory conditions that show diverse responses to IVIg treatment. We also intended to determine the targets of IVIg involved in the best treatment response of the evaluated diseases. Our selection and classification of diseases was based on a previously published systematic review, and we performed the disease characterization through manual curation of the literature. Furthermore, we undertook the mechanistic evaluation with artificial neural networks and pathway enrichment analyses. A set of 26 diseases was selected, classified, and compared. Our results indicated that diseases clearly benefiting from IVIg treatment were mainly characterized by deregulated processes in B cells and the complement system. Indeed, our results show that proteins related to B-cell and complement system pathways, which are targeted by IVIg, are involved in the clinical response. In addition, targets related to other immune processes may also play an important role in the IVIg response, supporting its wide range of actions through several mechanisms. Although B-cell responses and complement system have a key role in diseases benefiting from IVIg, protein targets involved in such processes are not necessarily the same in those diseases. Therefore, IVIg appeared to have a pleiotropic effect that may involve the collaborative participation of several proteins. This broad spectrum of targets and ‘non-specificity’ of IVIg could be key to its efficacy in very different diseases.

## Introduction

Intravenous immunoglobulin (IVIg) is a preparation of normal human IgG derived from large pools of healthy human plasma (1). IVIg is used as replacement therapy to prevent infections in patients with primary or secondary immune deficiency syndromes and, at high doses, exerts immunomodulatory effects to treat autoimmune and inflammatory disorders (2). Indeed, IVIg has been approved by the European Medicines Agency (EMA) for several autoimmune conditions, such as immune thrombocytopenia (ITP), chronic inflammatory demyelinating polyneuropathy (CIDP), Guillain-Barré syndrome (GBS), Kawasaki disease (KD), and multifocal motor neuropathy (MMN) (3). In addition, the off-label uses of IVIg are growing rapidly, and several other autoimmune and inflammatory conditions have been suggested as potential indications (4). Nonetheless, limited clinical evidence indicates that the successful response to IVIg observed in some autoimmune conditions may not necessarily and systematically be extended to others. For instance, adrenoleukodystrophy and inclusion body myositis do not respond well to this treatment (2, 5, 6).

The beneficial effect of IVIg is explained by mechanisms involving either its F(ab’)_2_ (7) or Fc domain (8) or both. The immunomodulatory effects of IVIg are complex and intricate; IVIg has been proved to modulate B and T cells, phagocytosis, complement activity, cytokine production, and the properties of dendritic cells (DCs), among many others (1, 9–11). This wide range of cellular and molecular targets can trigger pleiotropic effects in the immune system (1, 9). The specific immune and inflammatory processes modulated by IVIg depend on the pathophysiological process driving each disease (10, 12). Besides, the molecular mechanisms may also depend on the dose of IVIg and the window during the immunopathogenesis when IVIG is administered (13). Although IVIg has been shown to modulate a large number of immunological processes, the complete understanding of the molecular mechanisms defining treatment response remains unclear for some of them.

Systems biology and artificial intelligence tools have been used in the past to unveil the mechanism of action of a variety of drugs for hematological or cardiovascular diseases, among others (14, 15). However, this approach has been rarely used on IVIg, and the few reported examples were centered on the treatment of a specific disease (16–19). Therefore, we considered that the abovementioned tools could be applied in a larger setting to shed some light on the mechanisms of action of IVIg and their diversity depending on the treated disease. By means of system biology and machine learning models based on Artificial Neural Networks (ANNs) (20–22), we evaluated the differences in the pathophysiological pathways of autoimmune and inflammatory conditions that show diversity in responses to treatment with IVIg. Also, we aimed at determining the targets of IVIg involved in the best treatment response of the evaluated diseases.

## Materials and methods

### Selection and classification of diseases

To compare the heterogeneous efficacy response to IVIg treatment, we resorted to a previously published systematic and evidence-based classification of neurological and autoimmune diseases (2). We further classified the diseases according to their response to IVIg in four clusters, as previously reported (2): ‘definitely beneficial’ (DB), ‘probably beneficial’ (PB), ‘may provide benefit’ (MPB), and ‘unlikely to provide benefit’ (UPB) (**Table 1**, left column). The level and strength of evidence supported this classification according to: (i) evidence category, in groups Ia, Ib, IIa, IIb, III, and IV (e.g., group Ia referred to evidence obtained from meta-analysis of randomized controlled trials, while group IV to those obtained from expert committee reports, opinions or clinical experience of respected authorities of both), and (ii) strength of recommendation, which estimated the strength of the evidence to assign the condition to an IVIg response cluster, and ranged from A (strongest support) to D (weakest support) (2). For our study, we only included disorders for which the benefit/no benefit of IVIg treatment had been established with enough clinical evidence, i.e. those with a level of evidence Ia, Ib, IIa, or IIb (supported by clinical trials) and a strength of recommendation of A or B (2). We considered ‘IgM anti-myelin–associated glycoprotein (anti-MAG) paraprotein-associated peripheral neuropathy’ and ‘demyelinating neuropathy associated with monoclonal IgM’ as the same disease because of their degree of similarity and lack of differential molecular information available in scientific literature. Therefore, we hereafter referred to these two diseases as ‘IgM anti-MAG paraprotein-associated peripheral neuropathy’. To increase the number of analyzed disorders, we added two other autoimmune diseases with a well-established level of clinical response to IVIg: KD and Crohn´s disease (CD). IVIg has been approved by the US Food and Drug Administration (23) and the EMA (24) to treat KD according to strong evidence supporting its positive response (note that, in the abovementioned systematic review (2), KD was classified as an infection-related disease and not an autoimmune disease). Therefore, we incorporated KD into the DB cluster. On the contrary, unclear clinical evidence on the impact of IVIg on CD prompted us to include it in the UPB cluster in our study. We only reclassified one condition in our study, myasthenia gravis (MG), an approved indication for IVIg by the EMA. MG was moved from the PB to the DB cluster on the basis of the extended clinical use of IVIg in this condition and its positive response according to clinical evidence (25–27).

**Table 1:** Pathophysiological processes and number of effectors of evaluated neuroimmunological and autoimmune diseases according to reported response to IVIg.

IVIg response cluster	Disorders	Pathophysiological processes		Number of effectors
Definitely Beneficial (DB)	Chronic Inflammatory Demyelinating Polyradiculoneuropathy (CIDP)	2- T cell-mediated response		70
		1- Dysregulated B cell response		
		4- Complement system		
		3- Myelin damage by macrophages		
	Multifocal Motor Neuropathy (MMN)	1- Dysregulated B cell response		26
		4- Complement system	
		5- Myelin damage and axonal	
	Guillain-Barre Syndrome (GBS)	2- T cell-mediated response		67
		1- Dysregulated B cell response	
		4- Complement system	
		3- Myelin damage by macrophages	
	Graves ophthalmopathy (GO)	2- T cell-mediated response and inflammation by fibroblasts		52
		5- Orbital fibroblast proliferation and migration	
		1- Dysregulated B cell response	
		5- Production of ECM components by fibroblasts	
		3- Adipogenic and myofibroblastic differentiation	
	Immune thrombocytopenic purpura (ITP)	1- Dysregulated B cell response		61
		2- T cell-mediated response	
		4- Complement system	
		5- Suppression of megakaryocyte proliferation and maturation	
		5 - Dysfunctional mesenchymal stem cells (MSCs)	
	Kawasaki Disease (KD)	2- T cell-mediated response		95
		1- Dysregulated B cell response	
		4- Complement system	
		3- Exaggerated innate immune response - Systemic Inflammation	
		5- Aneurysm formation and angiogenesis	
	Myasthenia Gravis (MG)	1- Dysregulated B cell response		67
		2- T cell-mediated response	
		4- Complement system	
		5- Synaptic dysfunction	
		5- Muscular atrophy	
Probably Beneficial (PB)	Ig M anti-MAG paraprotein-associated peripheral neuropathy (anti-MAG IgM MGUS)	1- Dysregulated B cell response		22
		4- Complement system	
	Lambert-Eaton Myasthenic Syndrome (LEMS)	1- Dysregulated B cell response		29
		4- Complement system	
		5- Synaptic dysfunction	
		5- Muscular atrophy	
	Stiff-Person Syndrome (SPS)	1- Dysregulated B cell response		24
		2- T cell-mediated response	
		5- Synaptic dysfunction	
	Dermatomyositis (DM)	3- Exaggerated innate immune response		61
		2- T cell-mediated response	
		4- Complement system	
		5- Skin and muscle atrophy	
		1- Dysregulated B cell response	
Probably Beneficial	Birdshot retinochoroidopathy (BSRC)	2- Abnormal T cell activation		23
		2- T cell-mediated response	3- and inflammatory mediators’ perpetuation
	Henoch-Schonlein purpura (HSP)	3- Exaggerated innate immune response		48
		1- Dysregulated B cell response	
		2- T cell-mediated response	
		4- Complement system	
		5- Accelerated extracellular matrix breakdown	
May Provide Benefit (MPB)	Relapsing-Remitting Multiple Sclerosis (RRMS)	3- Exaggerated innate immune response		109
		2 -T cell-mediated response	
		1- Dysregulated B cell response	
		4- Complement system	
		5- Impaired neurotransmission	
	Intractable childhood epilepsy (ICE)	5- Drug-resistance		79
		5- Ion and neurotransmitter imbalance	
		3- Neuroinflammation by microglia and astrocytes	
	Postpolio syndrome (PPS)	3- Systemic inflammation		19
		5- Synaptic toxicity (induced by inflammatory mediators)	
		5- Muscular atrophy and inflammatory response	
	Juvenile idiopathic arthritis (JIA)	3- Exaggerated innate immune response		87
		2- T cell-mediated response	
		1- Dysregulated B cell response	
		3- NK cells dysfunction	
		2- Defective Tregs immunoregulation	
		4- Complement system	
		5- Joint damage	
	Anti-phospholipid antibody syndrome in pregnancy (APS)	5- Abnormal placental development		45
		3- Innate Immune Response	
		1- Dysregulated B cell response	
		4- Complement system	
		5- Thrombosis factor dysregulation	
	Severe rheumatoid arthritis (sRA)	2- T cell-mediated response		148
		1- Dysregulated B cell response	
		3- Synovial inflammation	
		5- Articular destruction	
		5- Bone erosion	
	Still disease (SD)	2- T cell-mediated response		63
		3- Exaggerated innate immune response	
		3- NK cells dysfunction	
	Felty’s syndrome (FS)	3- Exaggerated innate immune response		21
		1- Dysregulated B cell response	
		5- Neutropenia	
	Macrophage activation syndrome (MAS)	3- Exaggerated innate immune response		29
		2- T cell-mediated response	
		3- NK cells dysfunction	
		3- Cell death by activated macrophages	
Unlikely to Provide Benefit (UPB)	Polyarteritis nodosa (PAN)	3- Exaggerated innate immune response		28
		2- T cell-mediated response	
		2- Defective Tregs immunoregulation	
		5- Endothelial cells damage	
	Adrenoleukodystrophy (ALD)	5- VLCFA accumulation		61
		2- T cell-mediated response	
		5- Oxidative stress	
		3- Myelin damage by macrophages	
	Inclusion body myositis (IBM)	3- Exaggerated innate immune response		71
		2- T cell-mediated response	
		5- Muscle degeneration	
	Crohn's Disease (CD)	5- Intestinal barrier disruption		142
		3- Dysregulated intestinal immune response	
		2- T cell-mediated response	
		5- Tissue remodeling	

IVIg, Intravenous immunoglobulin.

**(Immune system) process grouping:** 
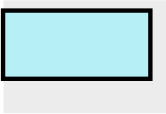
1- B cell-mediated processes 
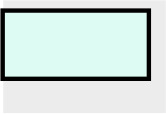
2- T cell -mediated processes 
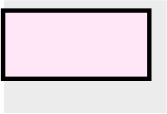
3- Innate immunity and inflammatory processes 
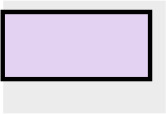
4- Complement system 
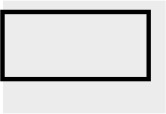
5- Other processes

### Disease characterization

We characterized the selected conditions molecularly through manual curation of the current scientific literature, as previously reported (21). First, we searched for reviews on the molecular pathogenesis, pathophysiology, and molecular mechanisms of the conditions in the PubMed database. Second, we retrieved the publications identified in these searches and assessed them at the title and abstract level. If molecular information describing the condition pathophysiology was present, we thoroughly reviewed full texts seeking to identify the main pathophysiological processes involved in the condition. We referred to these processes as ‘pathophysiological processes’. Third, we further characterized each pathophysiological process at the protein level (**Supplementary Table S1** in the **S1 File**). We reviewed the retrieved publications to identify protein/gene candidates to be condition effectors, i.e., proteins whose activity (or lack thereof) was functionally associated with the development of the condition. Finally, if we judged the evidence of the involvement of a candidate gene/protein in the condition not consistent enough for being considered an effector, we performed an additional PubMed search specifically on the protein candidate, including all protein names according to UniProtKB. The specific terms used for searches in the PubMed database and the abovementioned features for each studied disease are listed in **Supplementary Table S1** in the **S1 File**. We performed our searches in June 2020, limited them to 10 years before that date, and expanded them by reviewing relevant references in the results. We only included candidate articles with references more recent than year 2000, with the exception of references for anti-MAG, for which very little molecular information was found with this protocol, and for references for immunoglobulin heavy chain type detected among the autoantibodies in patients of each disease. The list of protein effectors considered for each disease is provided in **Supplementary Table S1** in the **S1 File**.

### IVIg targets characterization

To characterize the target profile of IVIg, we reviewed the Drugbank (28), Stitch (29), and Supertarget (30) data sources to obtain further information on IVIg targets. In addition, to completely characterize IVIg, we searched in the PubMed database on March 24, 2020, publications from the last ten years regarding known targets and mechanisms of action of IVIg, using the following main keywords in the title and/or abstract: “IVIG”, “Intravenous Immunoglobulin”, “Intravenous Immune Globulin”, “Intravenous Immunoglobulins”, “Intravenous Immune Globulins”, “Molecular”, “Mechanism”, “Pathophysiology”, “Pathogenesis”, “Mode”, “Action”, “Signaling”, “Signalling”, “Expression”, “Activation”, “Inhibition”, “Activity”. All results were evaluated, and reference lists were used to expand the search. Importantly, we considered IVIg targets only those proteins directly blocked or activated by IVIg (direct targets, **Table 2**). In contrast, we designated as ‘indirect targets’ those genes or proteins that, although lacking a direct interaction with IVIg, were modified by the effect of IVIg over its targets at the downstream level (**Table 3**). Finally, note that the artificial neuronal network (ANN) analysis performed in this work was focused solely on IVIg direct targets. Once identified, we classified direct targets in functional groups as per their immune-related function.

**Table 2:** List of IVIg protein targets (direct targets) identified by literature search and classified according to their related immunological function.

UniProt ID	Protein Name	Gene Name	Functional group	IVIg effect	Ref.
P20273	B-cell receptor CD22	CD22	B-cell related	↑	(31)
P08637	Low affinity immunoglobulin gamma Fc region receptor III-A	FCGR3A/CD16a	B-cell related	↓	(32)
O75015	Low affinity immunoglobulin gamma Fc region receptor III-B	FCGR3B/CD16b	B-cell related	↓	(32)
P12318	Low affinity immunoglobulin gamma Fc region receptor II-a	FCGR2A/CD32	B-cell related	↓	(32)
P55899	IgG receptor FcRn large subunit p51	FCGRT	B-cell related	↓	(33–35)
Q9Y275	Tumor necrosis factor ligand superfamily member 13B	TNFSF13B, BAFF	B-cell related	↓	(36–38)
O75888	Tumor necrosis factor ligand superfamily member 13	TNFSF13, APRIL	B-cell related	↓	(37)
Q9NYZ4	Sialic acid-binding Ig-like lectin 8	SIGLEC8	Antigen presentation	↑	(39)
Q9Y336	Sialic acid-binding Ig-like lectin 9	SIGLEC9	Antigen presentation	↑	(40)
P02745	Complement C1q subcomponent subunit A	C1QA	Complement system	↓	(41)
P00736	Complement C1r subcomponent chain	C1R	Complement system	↓	(42, 43)
P09871	Complement C1s subcomponent	C1S	Complement system	↓	(42, 43)
P01024	Complement C3	C3	Complement system	↓	(44–46)
P01031	Complement C5	C5	Complement system	↓	(47, 48)
P0C0L5	Complement C4-B	C4B	Complement system	↓	(46, 49)
–	HLA class I and II (includes 21 proteins)	HLA	HLA	↓	(50)
P25445	Tumor necrosis factor receptor superfamily member 6	FAS	Apoptosis	↑↓	(51)
Q9NNX6	CD209 antigen	CD209, DC-SIGN	Innate immunity	↑	(51–55)
Q9UMR7	C-type lectin domain family 4 member A	CLEC4A, DCIR	Innate immunity	↑	(56, 57)
P51681	C-C chemokine receptor type 5	CCR5	Innate immunity, T-cell related	↓	(58)
P0DSE2	M1-specific T cell receptor beta chain	TRB	T-cell related	↓	(59, 60)
P06127	T-cell surface glycoprotein CD5	CD5	T-cell related	↓	(61)
P01730	T-cell surface glycoprotein CD4	CD4	T-cell related	↓	(62)

HLA, Human leukocyte antigen; IVIg, Intravenous immunoglobulin; Ref., Reference.

↓, Inhibition; ↑, Activation.

**Table 3:** Indirect protein targets modulated by IVIg according to the literature.

Uniprot ID	Protein Name	Gene Name	Effect	Reference
P55774	C-C motif chemokine 18	CCL18	**↓**	(63)
P13500	C-C motif chemokine 2	CCL2	**↓**	(64)
P78556	C-C motif chemokine 20	CCL20	**↓**	(10)
P25942	Tumor necrosis factor receptor superfamily member 5	CD40	**↓**	(65, 66)
P33681	T-lymphocyte activation antigen CD80	CD80	**↓**	(67)
P42081	T-lymphocyte activation antigen CD86	CD86	**↓**	(67)
P46527	Cyclin-dependent kinase inhibitor 1B	CDKN1B	**↑**	(68)
Q9ULM6	CCR4-NOT transcription complex subunit 6	CNOT6	**↑**	(69)
P20023	Complement receptor type 2	CR2	**↓**	(68)
P04141	Granulocyte-macrophage colony-stimulating factor	CSF2	**↓**	(70)
P16410	Cytotoxic T-lymphocyte protein 4	CTLA4	**↑**	(69)
P31994	Low affinity immunoglobulin gamma Fc region receptor II-b	FCGR2B	**↑**	(65, 71)
Q9BZS1	Forkhead box protein P3	FOXP3	**↑**	(69)
P05362	Intercellular adhesion molecule 1	ICAM1	**↓**	(72, 73)
P01579	Interferon gamma	IFNG	**↓**	(71, 74)
P38484	Interferon gamma receptor 2	IFNGR2	**↓**	(75)
P22301	Interleukin-10	IL10	**↑**	(67, 70)
P29459	Interleukin-12 subunit alpha	IL12A	**↓**	(67)
P29460	Interleukin-12 subunit beta	IL12B	**↓**	(67)
P35225	Interleukin-13	IL13	**↑**	(76)
Q16552	Interleukin-17A	IL17A	**↓**	(10, 67)
Q96PD4	Interleukin-17F	IL17F	**↓**	(10, 67)
P01584	Interleukin-1 beta	IL1B	**↓**	(65, 67)
P18510	Interleukin-1 receptor antagonist protein	IL1RN	**↑**	(1)
P60568	Interleukin-2	IL2	**↓**	(71, 74)
Q9HBE4	Interleukin-21	IL21	**↓**	(10)
P08700	Interleukin-3	IL3	**↓**	(70)
O95760	Interleukin-33	IL33	**↑**	(57, 77)
P05112	Interleukin-4	IL4	**↑↓**	(57, 70, 77)
P05113	Interleukin-5	IL5	**↓**	(70)
P05231	Interleukin-6	IL6	**↓**	(72)
P20701	Integrin alpha-L	ITGAL	**↓**	(72)
P01374	Lymphotoxin-alpha	LTA	**↓**	(72)
P28482	Mitogen-activated protein kinase 1	MAPK1	**↑↓**	(1, 68)
Q15759	Mitogen-activated protein kinase 11	MAPK11	**↓**	(1)
P53778	Mitogen-activated protein kinase 12	MAPK12	**↓**	(1)
O15264	Mitogen-activated protein kinase 13	MAPK13	**↓**	(1)
Q16539	Mitogen-activated protein kinase 14	MAPK14	**↓**	(1)
P27361	Mitogen-activated protein kinase 3	MAPK3	**↑↓**	(1, 68)
P14780	Matrix metalloproteinase-9	MMP9	**↓**	(72)
P19838	Nuclear factor NF-kappa-B p105 subunit	NFKB1	**↓**	(1)
Q00653	Nuclear factor NF-kappa-B p100 subunit	NFKB2	**↓**	(1)
P35228	Nitric oxide synthase, inducible	NOS2	**↓**	(65)
P42336	Phosphatidylinositol 4,5-bisphosphate 3-kinase catalytic subunit alpha isoform	PIK3CA	**↓**	(68)
P42338	Phosphatidylinositol 4,5-bisphosphate 3-kinase catalytic subunit beta isoform	PIK3CB	**↓**	(68)
O00329	Phosphatidylinositol 4,5-bisphosphate 3-kinase catalytic subunit delta isoform	PIK3CD	**↓**	(68)
P48736	Phosphatidylinositol 4,5-bisphosphate 3-kinase catalytic subunit gamma isoform	PIK3CG	**↓**	(68)
P16885	1-phosphatidylinositol 4,5-bisphosphate phosphodiesterase gamma-2	PLCG2	**↓**	(68)
P35354	Prostaglandin G/H synthase 2	PTGS2	**↑**	(1, 78)
P51449	Nuclear receptor ROR-gamma	RORC	**↓**	(79)
P05109	Protein S100-A8	S100A8	**↓**	(80)
P06702	Protein S100-A9	S100A9	**↓**	(80)
P16581	E-selectin	SELE	**↓**	(72, 73)
P16109	P-selectin	SELP	**↓**	(73)
P40763	Signal transducer and activator of transcription 3	STAT3	**↓**	(1, 79)
P01137	Transforming growth factor beta-1 proprotein	TGFB1	**↑**	(65, 70)
P61812	Transforming growth factor beta-2 proprotein	TGFB2	**↑**	(65, 70)
Q9NYK1	Toll-like receptor 7	TLR7	**↓**	(1, 81)
Q9NR96	Toll-like receptor 9	TLR9	**↓**	(1, 67)
P01375	Tumor necrosis factor	TNF	**↓**	(71, 74)
Q9Y5U5	Tumor necrosis factor receptor superfamily member 18	TNFRSF18	**↑**	(69)
P43403	Tyrosine-protein kinase ZAP-70	ZAP70	**↑**	(75)

↓, Inhibition; ↑, Activation.

### Mechanistic evaluation of IVIg targets: Artificial neural network analysis

Therapeutic performance mapping system technology (14), based on systems biology, integrates available protein-protein network information along with physiological and pathophysiological data that allows to create machine learning models based on Artificial Neural Networks (ANNs), which have been previously defined and applied (20–22). ANNs are supervised algorithms that identify relations between proteins (e.g. drug targets) and clinical elements of the network (22, 82, 83) by inferring the probability of the existence of a specific relationship between two or more protein sets. The training set (**Supplementary Table S2** in the **S2 File**) was defined to predict the relationship between the drugs and their clinical conditions, drugs being stratified by the number of their of targets. The learning methodology used consisted in bagging training architecture of stratified ensembles of neural networks as final model. Each neural network model used was a multilayer perceptron (MLP) neural network classifier and they were considered as a weak classifier. These MLP were submitted to randomized initialization, having all of them a hidden layer containing between 7 and 11 nodes. Input database contained pairs of drug targets and biological conditions (drug-indication pairs) obtained from DrugBank and PubChem (84, 85). The final dataset was manually reviewed to avoid redundancies and mistakes that could affect the quality of the training set. The input feature vector is based on topological measures over the protein network from drug targets to condition according to the topological structure of the interconnection graph defined between them. The output was defined by a single node indicating the relation or no relation between the drug targets and the condition. Each training set for each MLP model was selected from a balanced subset of samples randomized by means of a Monte-Carlo cross-validation structure to reduce overfitting in the final model. The process to generate the final model was optimized by Levenberg-Marquardt strategy. A total of 1000 MLPs were trained and the best 75% of them for each stratum are considered in the final model. The implementation code was done in Matlab by using the Neural Network Toolbox, as combined with other Anaxomics Biotech’ developments. The final model performance is based on the capability to predict the pairs drug-indication, being evaluated as the AUC of the model. The final model has 81.77% of correct prediction when drugs tested have all their protein targets in the protein-protein network.

We employed these algorithms to explore the relationships between IVIg targets (either individually or grouped according to their functional group, **Table 2**) and the molecular definition of each disease (**Supplementary Table S1** in the **S1 File**). ANN analysis provides a score for a target, or group of targets, based on the validations of the prediction capacity of the mathematical models toward the training set (known drugs and diseases, as described in databases). Each score is associated with a p-value that describes the probability that the result is a true positive. Accordingly, and to simplify their interpretation, we divided here the ranking scores in four categories: strong relationship (ANN score >78%, p-value<0.05), medium-strong relationship (ANN score >71%, p-value<0.1), medium relationship (ANN score >38%, p-value<0.25), and low or no relationship (ANN score ≤38%, p-value ≥0.25).

### Data evaluation and statistical analysis

We used the chi-squared test to evaluate independence between the pathophysiological mechanisms involved in each disease and the disease classification based on IVIg response clustering. Also, we calculated inter-cluster disease similarity by direct protein overlap within the list of proteins included in the diseases of each cluster. Then, we performed the arithmetic mean of proportions of protein overlap (O) of each pair of disease clusters (C1, C2) according to equation 1:


$$\frac{O/C1+O/C2}{2}\times 100\;\;Equation\;1$$

We used the InteractiVenn software (86) to create Venn diagrams.

### Pathway enrichment analysis

We applied hypergeometric pathway enrichment analysis (87) to determine whether pre-defined protein sets, according to biological reference databases, were represented within groups of protein effectors involved in the IVIg response-based disease clusters. Specifically, we used KEGG (88) and Gene Ontology (89) as reference databases. Results were submitted to Benjamini-Hochberg multi-test correction (90) to control false discovery rate (FDR). We only selected enriched pathways with an FDR q-value<0.05.

## Results

### Comparison of IVIg responding and non-responding conditions at the pathophysiological level

A set of 26 autoimmune and inflammatory conditions, with clinical evidence of optimal level to be classified as responders/non-responders to IVIg, was selected. Previously reported IVIg response categories were used (2) (with a few exceptions, see Materials and Methods, **Table 1**). Once characterized, we compared the diseases at the level of pathophysiological processes to identify those processes more frequently related to each IVIg response cluster. To facilitate this comparison, we grouped them in broad (immune system) processes as follows (**Table 1**): T cell-mediated response, B cell-mediated response, complement system, innate immunity/inflammation processes, and other (which included disease-specific dysfunctions, such as bone erosion or muscular atrophy). This classification was based on the association of the proteins contained in each characterized pathophysiological process.

**Figure 1:**
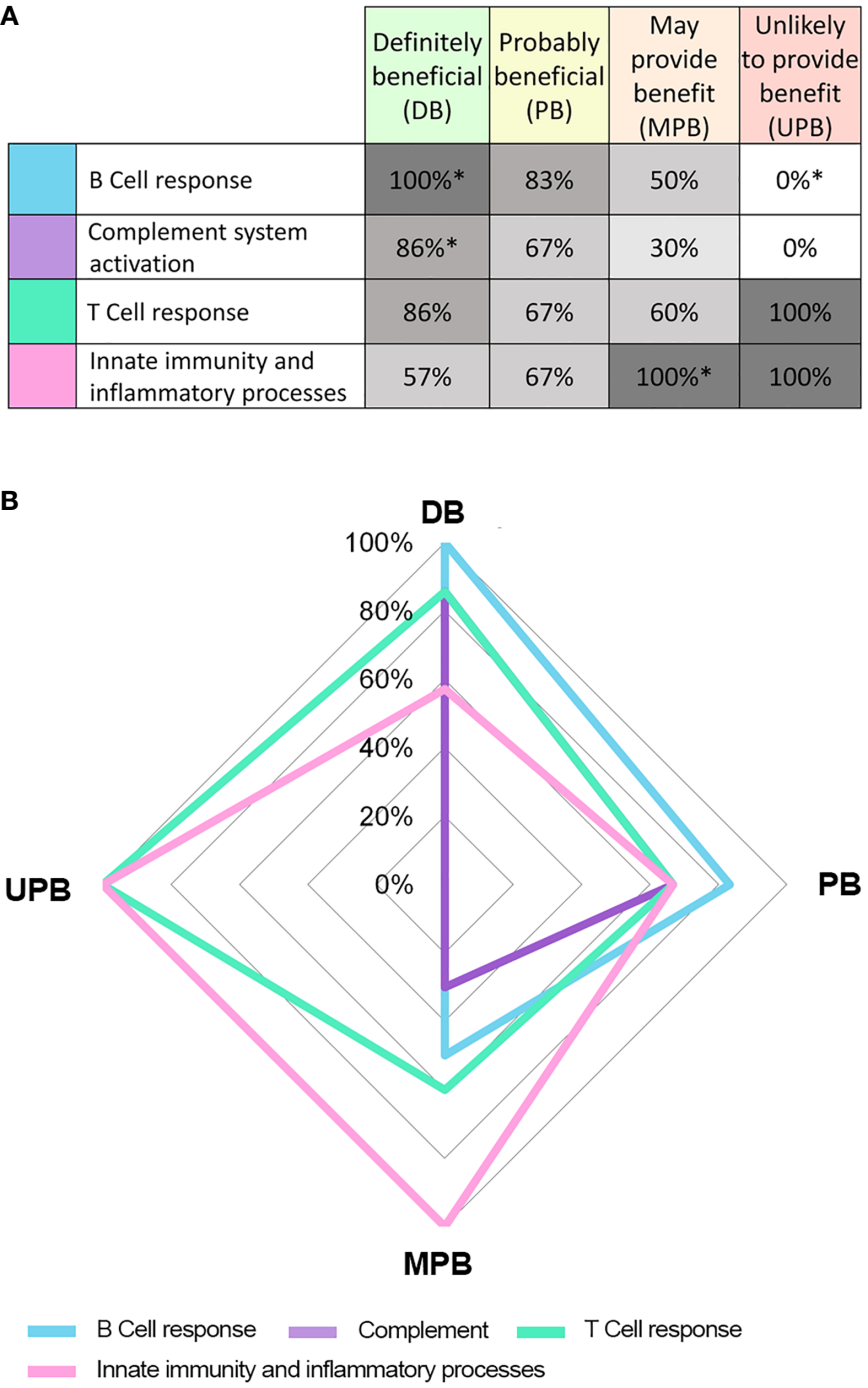
Results from the pathophysiological process grouping independence analysis with respect to different IVIg response clusters. Table **(A)** and graph **(B)** show the frequency (%) of conditions in each response cluster associated with each pathophysiological process. * Statistical significant (p< 0.05) result of the chi-squared test indicating dependence between the pathophysiological process group and cluster based in IVIg response.

Our results suggested that diseases within each IVIg response cluster shared similar pathophysiological processes, especially within the DB and UPB clusters, and that identifiable differences between the clusters could be found. A chi-squared test showed that there was dependency between pathological processes and disease classification based on IVIg response (**Figure 1**). In particular, diseases assigned to the DB cluster were positively associated with B cell-mediated processes and complement system. On the contrary, innate immunity and inflammatory processes were frequently associated to MPB (statistically significant association with 100% frequency) and UPB (not statistically association significant 100% frequency) clusters (**Figure 1**).

### Molecular pathways associated with diseases that clinically benefit from IVIg

**Figure 2:**
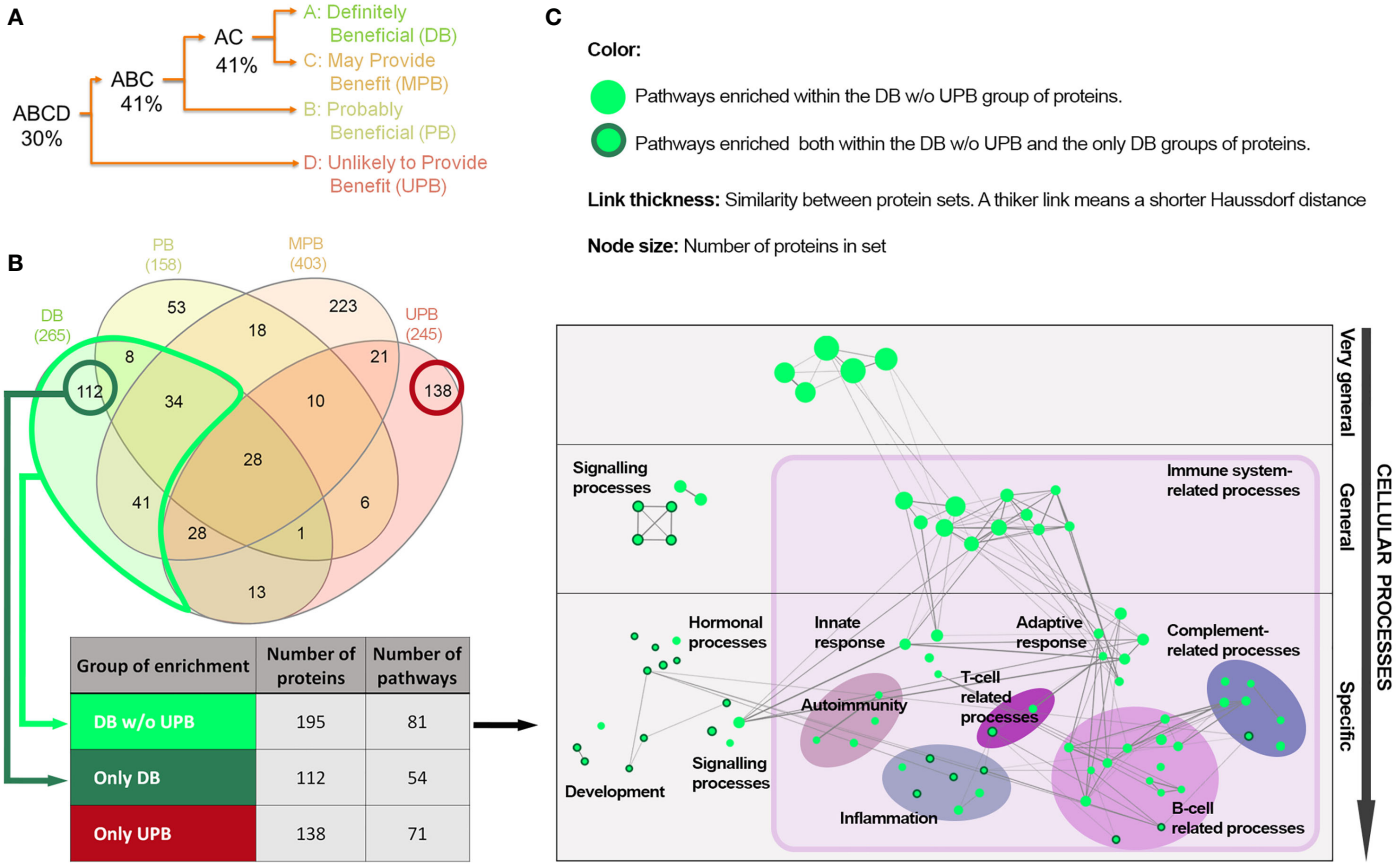
Protein and functional overlap between the IVIg response-based clusters of diseases. **(A)** Binary tree based on protein overlap among the disease clusters according to IVIg response. Percentages indicate the arithmetic mean of proportions of protein overlap of each pair of disease clusters or group of clusters in the tree branches; **(B)** Overlap of protein effectors between diseases with different response to IVIg (Venn diagram created with InteractiVenn (27)) and number of enriched pathways in the three protein sets defined according to the response-based clusters of diseases. the sets were defined in terms of number of proteins and enriched pathways according to the hypergeometric method (FDR q-value< 0.05); **(C)** Network representation of the 81 pathways enriched in the DB w/o UPB set of proteins (see **Table 3**) considering a Haussdorf distance< 1. IVIg: Intravenous immunoglobulin; only-DB: Protein effectors exclusively present in the IVIg ‘definitely beneficial’ cluster of diseases; DB w/o UPB: Protein effectors present in the IVIg ‘definitely beneficial’ cluster of diseases but not in the IVIg ‘unlikely to provide benefit’ cluster; only-UPB: Protein effectors exclusively present in the IVIg ‘unlikely to provide benefit’ cluster of diseases.

We evaluated similarity between the disease clusters based on IVIg response with the aim of identifying processes occurring in diseases that clinically benefit from IVIg. When analyzing direct protein overlap (**Figure 2A**) between the bibliography-based list of effectors (**Supplementary Table S1** in the **S1 File**) among the clusters, the DB and UPB clusters were the more distant. The specific effectors and those shared between the clusters were identified and grouped in three protein sets: proteins present in the DB cluster but not in the UPB cluster (DB w/o UPB), proteins specifically present in DB cluster (only DB), and proteins specifically present in UPB cluster (only-UPB) (**Figure 2B**). The enrichment analysis over the DB w/o UPB protein set provided 81 pathways enriched (**Figure 2B**). A detailed analysis of these enriched pathways (**Figure 2C**) unveiled that, while some of them were very general (e.g., positive regulation of the biological process, regulating signaling pathway), the most specific pathways pointed toward immune-related functions, such as processes of innate immunity response (e.g., leukocyte mediated immunity, phagocytosis), adaptive response (e.g., regulation of lymphocyte activation, adaptive immune response), autoimmunity (e.g., systemic lupus erythematosus, autoimmune thyroid disease), infection, T cell, B cell, and the complement system. Interestingly, there were other pathways enriched, including signaling processes (e.g., transferase activity, kinase activity, adrenergic signaling in cardiomyocytes) specific hormonal pathways (e.g., GnRH signaling pathway, thyroid hormone synthesis), and development pathways (e.g., progesterone-mediated oocyte maturation, long-term potentiation).

Taking the results of the enrichment analysis of the DB w/o UPB protein set as a reference, the results of the enrichment analysis of the only-DB protein set showed 23 common enriched pathways, while no overlap was found with the only-UPB set (**Supplementary Table S3** in the **S3 File**). The 23 enriched pathways shared between the only-DB and DB w/o UPB sets contained mainly general signaling pathways involved in development (e.g., oocyte meiosis and regulation of dendritic differentiation), hormonal regulation (e.g., endocrine-regulated calcium reabsorption and GnRH signaling pathway), and inflammation (e.g., VEGF and cGMP-PKG signaling pathways), as well as some pathways related to B and T cells (**Figure 2C**, nodes circled in dark green, and **Supplementary Table S3** in the **S3 File**).

### Targets involved in the response to IVIg

**Figure 3:**
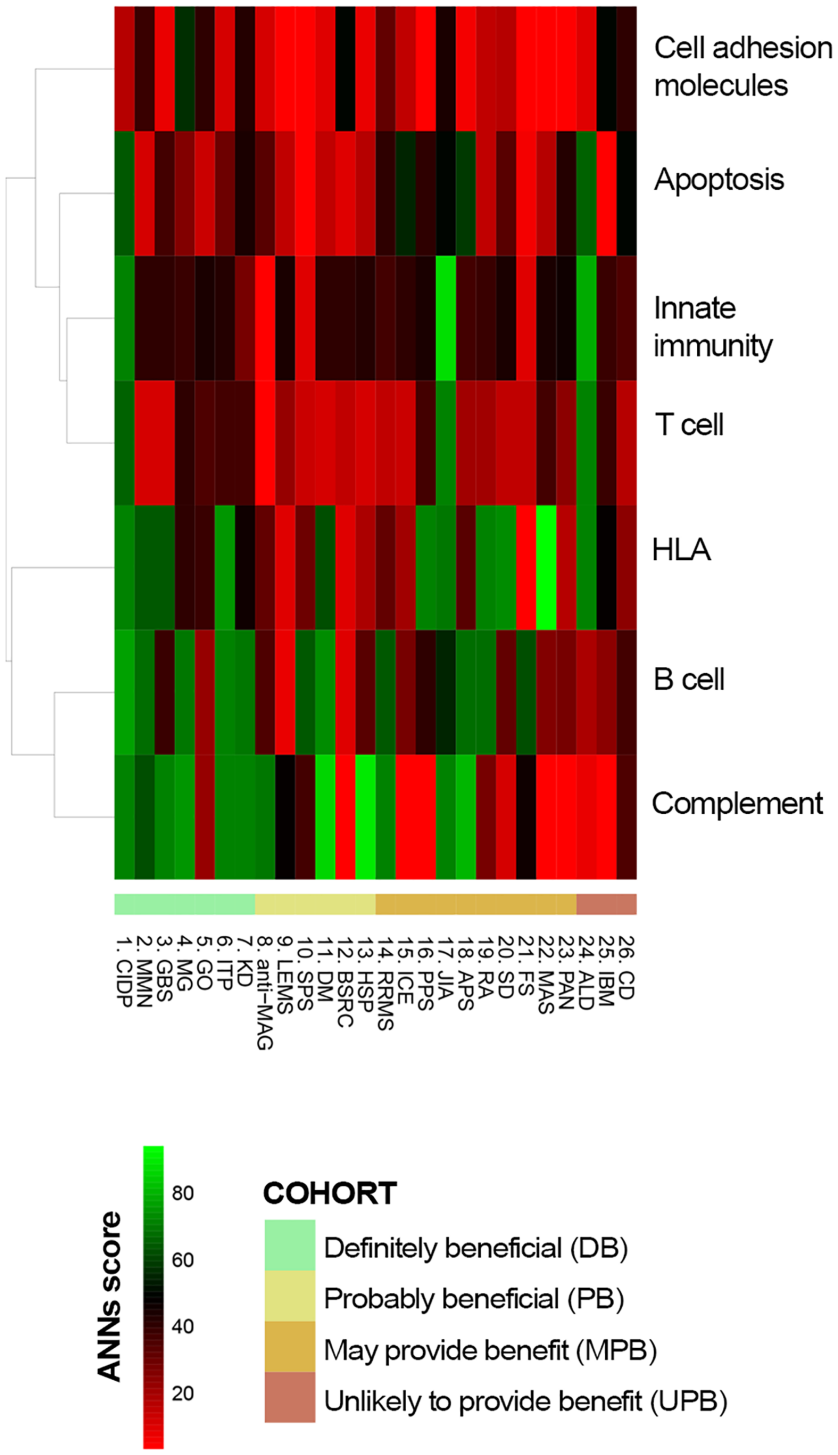
Predicted relationship between each studied immune-related disorder and each pathophysiological process affected by IVIg according to ANN. ANN, Artificial Neural Network; IVIg, Intravenous immunoglobulin.

**Figure 4:**
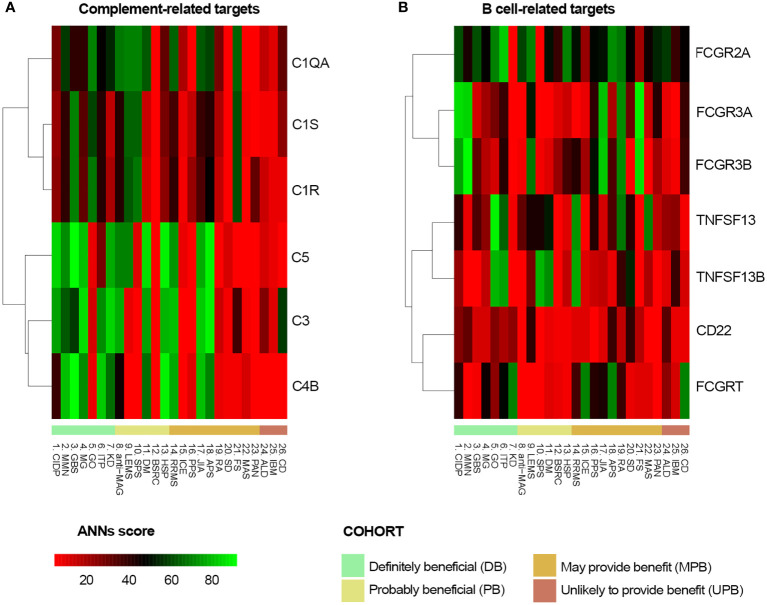
Predicted relationship between each studied disorder and **(A)** each complement system protein target of IVIg or **(B)** each B-cell related IVIg protein target according to ANN. ANN, Artificial Neural Network; IVIg, Intravenous immunoglobulin.

The functional relationship between IVIg targets (**Table 2**) and the different diseases, defined as the effector proteins previously identified (**Supplementary Table S1** in the **S1 File**), was tested by ANN analysis to measure the mechanistic relationship between them. This approach predicts the possibility that the studied targets modulate the set of proteins involved in the disease pathophysiology. We first tested the functional relationship between the studied conditions and IVIg targets grouped by their immune-related function (*Functional group* column in **Table 2**). The results showed that B cell-related and complement system-related targets were more likely associated with IVIg efficacy (**Figure 3**). To get a closer evaluation of the targets, the analysis was performed for each of them individually, focusing on the targets within the complement system and B cell-related functional groups. The analysis over the individual IVIg targets of the complement system functional group disclosed a stronger association between C3, C4B, and C5 with diseases assigned to the DB cluster than their association with disorders classified in the UPB cluster (**Figure 4A**). The same evaluation performed on individual targets related to B cells showed that different groups of targets were associated with different diseases (namely, FCGR3A/FCGR3B and TNFSF13/TNFSF13B) (**Figure 4B**).

The participation in IVIg response of other targets not related to B cell or complement system processes was also explored by ANNs. In this case, the functional relationship between the complete IVIg protein target profile and each disease of the DB cluster was assessed and unveiled a high heterogeneity among diseases (**Table 4**). Targets associated with HLA (e.g., HLA-DPA1), innate immunity (e.g., CLEC4A), or T-cell related (e.g., CCR5) showed a consistent medium-strong (>71% ANN score) or strong (>78% ANN score) relationship for all diseases (except for MMN) and could be involved in the treatment’s efficacy. Results in **Table 4** also reinforced the observation that the contribution of each B cell-related target differed for each condition. The response to IVIg in GBS, MG, and graves ophthalmopathy (GO) could not be attributed to individual B cell-related targets (all of them presented ANN scores below 71%), but complement system-related targets seemed to have a potential individual role (showing at least 71% ANN score; e.g., C5 for all diseases except GO and ITP, and C4B for all diseases as well, except for CIDP, GO, and KD).

**Table 4:** Functional relation (ANN score) between IVIg protein targets and diseases from the DB cluster according to ANN analysis.

	CIDP	MMN	GBS	MG	GO	ITP	KD
FAS (P25445) *Apoptosis*	++ (72%)	+ (64%)	+ (64%)	+ (41%)	+ (39%)	++ (74%)	+ (46%)
FCGR3A (P08637) *B cell-mediated*	**+++** **(86%)**	**+++** **(85%)**	– (11%)	+ (52%)	– (20%)	– (27%)	– (37%)
FCGR3B (O75015) *B cell-mediated*	++ (76%)	**+++** **(93%)**	– (31%)	+ (69%)	– (17%)	– (12%)	+ (43%)
FCGR2A (P12318) *B cell-mediated*	+ (62%)	+ (44%)	+ (61%)	+ (71%)	+ (47%)	+ (71%)	**+++** **(83%)**
TNFSF13 (O75888) *B cell-mediated*	+ (40%)	– (8%)	– (35%)	+ (44%)	– (24%)	**+++** **(90%)**	+ (66%)
TNFSF13B (Q9Y275) *B cell-mediated*	– (22%)	– (3%)	– (5%)	– (28%)	– (15%)	**+++** **(79%)**	++ (76%)
SIGLEC9 (Q9Y336) *Antigen presentation*	– (14%)	– (16%)	– (20%)	– (18%)	++ (73%)	– (14%)	– (34%)
C5 (P01031) *Complement system*	**+++** **(86%)**	++ (72%)	**+++** **(93%)**	**+++** **(82%)**	– (9%)	– (27%)	++ (77%)
C3 (P01024) *Complement system*	++ (77%)	+ (63%)	+ (59%)	**+++** **(86%)**	– (11%)	++ (72%)	**+++** **(85%)**
C4B (P0C0L5) *Complement system*	– (38%)	**+++** **(82%)**	**+++** **(92%)**	++ (72%)	– (7%)	**+++** **(84%)**	+ (67%)
C1QA (P02745) *Complement system*	– (28%)	+ (58%)	+ (41%)	+ (40%)	++ (71%)	+ (47%)	+ (54%)
C1R (P00736) *Complement system*	– (23%)	+ (40%)	++ (71%)	– (32%)	+ (68%)	+ (41%)	– (18%)
C1S (P09871) *Complement system*	– (22%)	+ (40%)	++ (71%)	– (33%)	+ (60%)	+ (42%)	– (15%)
HLA-DPA1 (P20036) *HLA*	**+++** **(80%)**	+ (68%)	**+++** **(81%)**	+ (42%)	– (36%)	++ (72%)	+ (41%)
HLA-DQA1 (P01909) *HLA*	++ (76%)	+ (41%)	+ (56%)	++ (73%)	– (36%)	+ (71%)	+ (39%)
HLA-DRA (P01903) *HLA*	++ (72%)	+ (44%)	+ (50%)	+ (39%)	++ (71%)	+ (49%)	– (33%)
HLA-DPB1 (P04440) *HLA*	++ (71%)	– (22%)	– (9%)	– (36%)	– (37%)	+ (51%)	– (18%)
HLA-B (P01889) *HLA*	+ (51%)	– (23%)	– (26%)	+ (46%)	– (26%)	– (30%)	++ (71%)
CLEC4A (Q9UMR7) *Innate immunity*	++ (73%)	+ (65%)	++ (71%)	++ (72%)	++ (72%)	++ (72%)	++ (72%)
CD209 (Q9NNX6) *Innate immunity*	++ (71%)	– (3%)	+ (63%)	+ (71%)	++ (71%)	+ (57%)	++ (73%)
CCR5 (P51681) *Innate immunity, T cell-related*	**+++** **(93%)**	– (36%)	+ (67%)	+ (39%)	+ (69%)	+ (66%)	+ (43%)
CD4 (P01730) *T cell-related*	++ (71%)	+ (50%)	**+++** **(87%)**	+ (63%)	– (38%)	+ (40%)	+ (57%)

ANN scores represent the probability for the relationship to be a true positive: +++ (in bold) correspond to a strong relationship and values > 78% (p-value <0.05); ++ correspond to a medium-strong relationship and values > 71% (p-value <0.1); + correspond to a medium relationship and values >38% (p-value <0.25); and – correspond to low or no relationship and values ≤ 38% (p-value ≥0.25).

ANN, Artificial Neural Network; CIDP, Chronic inflammatory demyelinating polyradiculoneuropathy; DB, Definitely beneficial; GBS, Guillain-Barre syndrome; GO, Graves ophthalmopathy; HLA, Human leukocyte antigen; ITP, Immune thrombocytopenic purpura; IVIg, Intravenous immunoglobulin; KD, Kawasaki disease; MG, Myasthenia gravis; MMN, Multifocal motor neuropathy.

Only targets with a strong relationship with at least one disease are shown. Proteins are shown as gene name (Uniprot ID) and related functional group.

## Discussion

Our *in silico* systems biology-based approaches, described here, aimed to explore and compare the pathophysiology of autoimmune diseases with different degrees of clinical response to IVIg treatment. They also addressed IVIg’s mechanisms of action on those disorders responding to IVIg treatment. The results obtained allowed us to differentiate the main pathophysiological processes behind each of the IVIg response-based clusters of diseases analyzed and to suggest which IVIg targets could be involved in the successful response to this treatment.

The classification chosen here as the basis of the study (2) has been repeatedly used in recent publications (91–93), thus, supporting our election and findings. Our results showed that diseases clearly benefiting from IVIg therapy (i.e., belonging to the DB cluster) were mainly characterized by B-cell and complement system-related processes. However, our analyses did not rule out the role of other processes that could be relevant to these diseases. Furthermore, current treatments for the conditions in the DB cluster support the important role of B cell and complement system processes. B cell depletion has been stablished as a relevant target for autoimmunity disorders for long (94). For example, rituximab is a B-cell depleting monoclonal antibody that, despite not being approved for the treatment of neurological disorders, has shown a certain degree of clinical improvement in many diseases included in our DB cluster (i.e., CIDP, MMN, GO, MG) (95, 96). Similarly, eculizumab is a monoclonal antibody that inhibits terminal complement activation by binding to C5 (90); it is approved to treat MG (97) and has also been successfully used in patients with MMN (96). These examples would support the key role of B-cell and/or complement system processes in these conditions, hence, in agreement with our findings.

On the contrary, our results indicated that a minor response to IVIg treatment was likely in pathologies in which B cells were not predominantly involved. It is clear that T cell populations play a critical role in mediating autoimmunity *via* T cell inflammatory cytokine secretion, their help to B cells *via* cytokines thus driving the naïve B cells to become autoantibody secreting plasma cells. Interestingly, literature evidence indicates that IVIg modulates T cell-related processes (65, 67) and innate immunity/inflammation (1, 98). Although our results do not support that pathological alterations in these processes determine the level of response to IVIg in a specific disease, they however endorse a role for T cell- and inflammation-related IVIg targets in its therapeutic effect for some of the evaluated diseases.

When evaluating which IVIg targets might have a direct role over B cell and complement system processes through ANN analyses, we found a relevant role for the complement proteins C3, C4B, and C5 in IVIg mechanisms. In fact, it has been described that IVIg directly neutralizes these proteins through its F(ab’)_2_ region (44), and through the formation of complexes between IVIg and complement components (99). This neutralization mechanism would explain the impact on complement activation in several of the analyzed diseases, which in turn prevents complement-mediated tissue damage (100). Evidence supports the relevance of complement activation inhibition by IVIg in the therapeutic efficacy in autoimmune dermatological conditions, reinforce the relevance of this mechanism within IVIg therapeutic effects (101). In contrast, no common B cell-related targets were found, probably due to the great complexity of B cell pathological role in autoimmune conditions (e.g., autoantibodies production, cytokines release, autoantigens presentation to T cells) (102). Previous reports have proven that IVIg is able to modulate B-cell function and survival (103, 104). In agreement with these facts, our models showed that modulation of B-cell pathways by IVIg might involve different targets for each disease. These results are also in line with previous findings reporting that IVIg contains antibodies against a proliferation-inducing ligand and, more importantly, against B-cell activating factor involved in B-cell survival and with documented deleterious effects in B-cell mediated autoimmune diseases (36, 37). Furthermore, the survival and function of B cells can be significantly impacted by the anti-idiotypic antibodies present in IVIg (103, 105). Also, it has been reported that the anti-CD5 antibodies found in IVIg can inhibit the release of autoantibodies produced by B cells (106).

While not directly addressed in the ANN analysis (which focused on protein targets independently of the cell where they are expressed), evidence points to the role of different cell types in IVIg-mediated immunomodulation. In this sense, DCs can regulate immune responses through interaction with T and B lymphocytes and have been demonstrated to be targets of the immunomodulatory effects of IVIg (107, 108). This has been observed particularly through the inhibition of CD80 and CD86 expression (69) and by promoting a more tolerogenic phenotype (109) that is less competent in driving lymphocyte proliferation (65, 67). Furthermore, IVIg has been found to modulate DCs functions through both FcγR- and non-Fc-receptor-mediated signaling events (110). In contrast, even though DCs regulate B cell function, the effects of IVIg over DCs do not seem to affect B cells directly (111). IVIg has been reported to affect directly and indirectly Th17 and Treg function (69, 79, 112–115), and reciprocally regulate them. Treg function is central in the maintenance of immune tolerance and has been found to be reduced in patients suffering from autoimmune diseases (116). IVIg treatment has been shown to promote development or activation of Treg through diverse mechanisms (113, 117). For example, interaction of IVIg with DCs through binding of sialylated IVIg to C-type lectin receptors that induces inhibitory FcγrIIb expression on DC (56). This renders the DC tolerogenic and leads to a reduced costimulatory molecule expression and proinflammatory cytokine secretion (118), thus favoring Treg function. However, whether Fc-sialylation is critical for IVIG-mediated Treg cell expansion in humans is still unclear (117). It has also been shown that, upon IVIg treatment, DC-SIGN (dendritic cell-specific intercellular adhesion molecule-3-grabbing nonintegrin) signaling is able to expand Treg through prostaglandin E2 secretion in DCs (119, 120). In addition, IVIg can also modulate the production of proinflammatory cytokines by DC, which may play a role in maintaining T-cell tolerance (69). De Groot and coworkers (121) described a DC-dependent mechanism where promiscuous IgG-derived T-cell epitope peptides (Tregitopes) increases pro-tolerogenic cytokine production (IL-10) and converts naïve conventional T cells to T regulatory cells (122). Treg would then inhibit effector Th1, Th2 and Th17 cellular activity in inflammatory microenvironments and secrete anti-inflammatory cytokines (112, 123–125). Overall, the presence of Tregitopes might in part explain the success of IVIg therapy in treating autoimmune diseases (122, 126). IVIg treatment has been reported to affect natural killer cells and subsequently regulate Treg function in KD patients (127). IVIg has been shown to affect T cells chemokine production, as observed by Pigard and colleagues, thus affecting their function and compartmentalization (128). Our results position the modulation of B cells as an outstanding IVIg therapeutic mechanism. In fact, aside from antibody producers, B cells also act as APC and cytokine producing cells (129). This role of B cells as APC in regulating T cell equilibrium, Treg induction and establishment of tolerogenesis must have contributed to these findings (130–132).

Basophils have also been shown to be activated when treated with IVIg in mouse models and human studies (133–136) through indirect mechanisms that our approach was not able to discern. Notwithstanding their importance, the indirect nature of the effect and the uncertainty of the direct molecular target of IVIg over these cell types might have resulted in overlooking these mechanisms in our ANN analysis. However, these mechanisms were not highlighted either as pathologic drivers characterizing the diseases within the DB disease cluster.

In the last decade, research and development have surged on novel molecules as potential therapeutic alternatives to IVIg, aiming to overcome IVIg’s therapeutic limitations (e.g., dependence on the supply of human plasma and the large doses required) (137). These promising new therapies belong to the group of next-generation Fc receptor-targeting biologics (138): (i) Recombinant fragment crystallizable (rFc) multimers, designed to have multiple, organized, and structured IgG-Fc moieties; (ii) Neonatal Fc receptor (FcRn)-targeting therapeutics; and (iii) Fc/Fcγ receptor (FcγR)-targeting therapeutics (137). Importantly, FcRn-targeting compounds should be highlighted since our results did not suggest a major relevance of this IVIg target to achieve a response. The FcRn blocker efgartigimod is currently the compound furthest advanced in clinical trials (139–142), with some of the anti-FcRn monoclonals following not far behind (143, 144), while a modified monomeric recombinant Fc optimized for binding to all FcRns and FcγRs is also under development (145). This search for novel molecules specifically targeting Fc receptors contrasts with the results obtained in our study, which suggest that the ‘non-specificity’ of IVIg and its interaction with a broad spectrum of targets could be key to obtaining a pleiotropic effect and clinical efficacy in very different diseases. Regarding Fc multimers, the activity of these molecules relies not only on the interaction with Fc receptors but also on targeting complement proteins (146). The comparison of the activity of these new drugs with IVIg would be interesting since some of the direct targets of IVIg herein analyzed interact with IgG’s Fab region, such as CD209/DC-SIGN, C3a, and C5a anaphylotoxins (proteolytic degradation products of C3 and C5) (147, 148).

Our study was limited by the intrinsic constraints of systems biology–based modeling approaches, which include issues on information availability about drugs and diseases. First, our results were influenced by the list of diseases included in the study and the IVIg response cluster assigned to each of them. We selected only autoimmune and neuroautoimmune conditions with the strongest clinical evidence (i.e., tested in clinical trials) for their further classification, including only those tested in clinical trials. Thus, some current ‘off-label’ uses of IVIg were not considered here, as is the case of blistering autoimmune diseases (e.g., systematic lupus erythematosus, pemphigus or pemphigoid diseases). IVIg treatment has been shown to benefit patients suffering from these diseases (149–151), and is usually used in a second- or third-line setting for these conditions (77). However, the level of evidence obtained to date, according to the criteria set in the current study, prevented the inclusion of these diseases in the analysis. Of note, many of these conditions are rare and, even if clinical trials had been conducted, the small sample size could have been considered a drawback. This could have precluded their clear classification, thus finishing in the MPB or PB clusters. Consequently, future clinical evidence regarding IVIg use could modify our classification, increasing the pool of available indications in the top and bottom clusters (i.e., DB and UPB). Second, the characterization of the studied diseases may have been incomplete since many of them were rare or complex diseases in which only a few research teams are actively working at the molecular level, potentially leading to biased literature (we found less than 25 effector proteins for several characterized diseases, for instance: Stiff-person syndrome, birdshot retinochoroidopathy, postpolio syndrome, and Felty’s syndrome, see **Table 1**). This limitation might have affected the characterization and analyses at the protein level, perhaps with a weaker impact on the definition of the pathophysiological processes. Finally, although we reviewed all available information at the time of the study for identification of direct IVIg targets, including FcγR and non-receptor protein targets, the extreme complexity of IVIg, due to its multi-target feature at the molecular and cellular level, could have led us to underestimate the potential impact of IVIg by including an incomplete list of targets because of a dearth of evidence. Indeed, a recent study by Pipi *et al.* (2021) (101) unraveled potential non-receptor-mediated antioxidative mechanism for high-dose IgG, as neutrophil elastase substrate and through ROS scavenging, potentially involved in the treatment of skin autoimmunity; however, for the latter they did not detail the mechanism nor specified the direct IVIg protein target, which was a requirement for our approach. Also, considering only direct IVIg targets might have oversimplified the model, hindering the detection of effects over immune components indirectly affected by IVIg; and categorizing the identified targets in discrete function compartments, which might have oversimplified the IVIg mechanisms explored in the ANN analysis on overall processes.

However, using systems biology–based modeling approaches minimizes the impact of the potential biases intrinsic to the availability of information. These models compile and reinterpret available biological data to generate new knowledge and hypotheses while reproducing known aspects of the diseases or drugs. Our models were built considering the whole human protein network and a wide range of drug-pathology relationships, not only limited to the studied diseases or inflammatory conditions, presenting cross-validation accuracies above 80%.

## Conclusion

Systems biology approaches combined with machine learning are becoming increasingly important for identifying new drug effects and disease mechanisms. Using these techniques, we dedicated our work to compile all available information in order to gain a better understanding of IVIg mode of action. In our analyses, diseases clearly benefiting from IVIg treatment (i.e., the DB cluster) were found to be mainly characterized by deregulated processes in B cells and the complement system. In addition, IVIg targets related to B-cell and complement system pathways seemed to be involved in the clinical response. However, targets related to other immune processes may also play an important role in the IVIg response, supporting its wide range of action through several mechanisms. Besides, although B-cell responses and complement system have a key role in diseases benefiting from IVIg, protein targets involved in such processes are not necessarily the same in those diseases. Our results support further investigations on the role of IVIg in diseases where B cells and the complement system are relevantly involved and for which no evidence has been gathered yet or is inconclusive, for instance, those classified here as PB and MPB. Finally, since the level of relationship varied between proteins included in the DB cluster and different diseases, IVIg appeared to have a pleiotropic effect that may involve the collaborative participation of several targets. Indeed, the weight of each target in treatment efficacy may be different for each condition. Therefore, IVIg’s broad spectrum of targets and ‘non-specificity’ could be key to its efficacy in very different diseases.

## Data availability statement

The datasets presented in this study can be found in online repositories. The names of the repository/repositories and accession number(s) can be found in the article/**Supplementary Material**.

## Author contributions

JT, EC-G, SC, and SK have significantly contributed to setting up fundamental questions about IVIg. JT, EC-G, SC, and TS contributed to conceptualize the study. TS and CS-V contributed to study design. EC-G, SC, and TS contributed to supervise and manage the project. CS-V and HB contributed to formal analysis, data curation, and visualization. CS-V contributed to the methodology and investigation. SK contributed to the interpretation of data. CS-V drafted the manuscript and the rest of authors contributed to the critical revision of the text. All authors have given their final approval to the version submitted for publication.

## Acknowledgments

The authors thank Dirk Büscher for his valuable contributions to the conceptualization of the study and Matías Rey (BCN Medical Writing, Spain) for his assistance in writing and copyediting the manuscript.

## Conflict of interest

EC-G, SC, and JT are full time employees at Grifols. CS-V and HB are full time employees at Anaxomics Biotech. TS was a full time-employee at Anaxomics Biotech at the time of the study. SK has received fees for lectures and reviewing research proposals from CSL-Behring and Grifols.

The authors declare that this study received funding from Grifols (including medical writing assistance, and the article processing fees). The funder participated in the original idea, writing of this article and decision to submit it for publication, but was not involved in the study design, collection, analysis and interpretation of data.

## Publisher’s note

All claims expressed in this article are solely those of the authors and do not necessarily represent those of their affiliated organizations, or those of the publisher, the editors and the reviewers. Any product that may be evaluated in this article, or claim that may be made by its manufacturer, is not guaranteed or endorsed by the publisher.
