# Supplementary material for: Systems biology and artificial intelligence analysis highlights the pleiotropic effect of IVIg therapy in autoimmune diseases with a predominant role on B cells and complement system
Source: Front Immunol. 2022 Sep 30;13:901872. doi: 10.3389/fimmu.2022.901872 (PMC9563374; doi:10.3389/fimmu.2022.901872)
Supplement: Supplementary file 2 [file Table_2.docx]

***Supplementary Material S2 File***

**Supplementary table:**

- **Supplementary Table S2**

| **Supplementary Table S2. Summary of data used for model construction (network and training set)** | |
| --- | --- |
| **Data type** | **Number of entries** |
| **In-house databases information** | |
| Considered Interactions | 437 955 |
| Considered Proteins | 17 081 |
| Characterized Drugs | 1 073 |
| Drug Targets | 2 690 |
| Characterized Clinical Conditions | 168 |
| Clinical Conditions Key Proteins Characterized | 4 076 |
| **Training set information** | |
| Curated drug-indications restrictions | 180 264 (1 731positive) |
| Drug-ADRs restrictions | 30 096 (2 640 positive) |
| Drug-indications/ADRs protein correlations | 2 655 |
| ADR: Adverse drug reaction | |
